# A Novel Egr-1-Agrin Pathway and Potential Implications for Regulation of Synaptic Physiology and Homeostasis at the Neuromuscular Junction

**DOI:** 10.3389/fnagi.2017.00258

**Published:** 2017-08-03

**Authors:** Ryen MacDonald, Sebastien Barbat-Artigas, Chulmin Cho, Huashan Peng, Jijun Shang, Ayman Moustaine, Salvatore Carbonetto, Richard Robitaille, Lorraine E. Chalifour, Hemant Paudel

**Affiliations:** ^1^Lady Davis Institute for Medical Research, Jewish General Hospital Montreal, QC, Canada; ^2^Integrated Program in Neuroscience, McGill University Montreal, QC, Canada; ^3^Département de neurosciences, Université de Montréal Montreal, QC, Canada; ^4^Center for Research in Neuroscience Montreal, QC, Canada; ^5^Department of Medicine, McGill University Montreal, QC, Canada

**Keywords:** agrin, neuromuscular junction, aging, physiological processes, homeostasis, synapses, signaling pathway

## Abstract

Synaptic transmission requires intricate coordination of the components involved in processing of incoming signals, formation and stabilization of synaptic machinery, neurotransmission and in all related signaling pathways. Changes to any of these components cause synaptic imbalance and disruption of neuronal circuitry. Extensive studies at the neuromuscular junction (NMJ) have greatly aided in the current understanding of synapses and served to elucidate the underlying physiology as well as associated adaptive and homeostatic processes. The heparan sulfate proteoglycan agrin is a vital component of the NMJ, mediating synaptic formation and maintenance in both brain and muscle, but very little is known about direct control of its expression. Here, we investigated the relationship between agrin and transcription factor early growth response-1 (Egr-1), as Egr-1 regulates the expression of many genes involved in synaptic homeostasis and plasticity. Using chromatin immunoprecipitation (ChIP), cell culture with cell lines derived from brain and muscle, and animal models, we show that Egr-1 binds to the *AGRN* gene locus and suppresses its expression. When compared with wild type (WT), mice deficient in Egr-1 (Egr-1−/−) display a marked increase in *AGRN* mRNA and agrin full-length and cleavage fragment protein levels, including the 22 kDa, C-terminal fragment in brain and muscle tissue homogenate. Because agrin is a crucial component of the NMJ, we explored possible physiological implications of the Egr-1-agrin relationship. In the diaphragm, Egr-1−/− mice display increased NMJ motor endplate density, individual area and area of innervation. In addition to increased density, soleus NMJs also display an increase in fragmented and faint endplates in Egr-1−/− vs. WT mice. Moreover, the soleus NMJ electrophysiology of Egr-1−/− mice revealed increased quantal content and motor testing showed decreased movement and limb muscle strength compared with WT. This study provides evidence for the potential involvement of a novel Egr-1-agrin pathway in synaptic homeostatic and compensatory mechanisms at the NMJ. Synaptic homeostasis is greatly affected by the process of aging. These and other data suggest that changes in Egr-1 expression may directly or indirectly promote age-related pathologies.

## Introduction

Chemical synapses allow organized, directional communication between two neurons, or between a neuron and a non-neuronal contact, such as a muscle cell at the neuromuscular junction (NMJ). Neuronal circuitry is dynamically shaped and tuned by diverse processes of plasticity in order to maintain stability and function (Changeux and Danchin, 1976; Nelson and Turrigiano, 2008). Damage to any component of synaptic machinery can lead to loss of synaptic stability, disruption of neuronal circuitry and subsequent neurological or neuromuscular disease (van Spronsen and Hoogenraad, 2010).

Robust homeostatic control governs synaptic activity at the vertebrate NMJ (Frank, 2014). Synaptic homeostasis at the NMJ allows the pre- and/or postsynaptic machinery to adapt (Turrigiano, 2007; Davis and Müller, 2015), and maintain function in response to a change in properties of either presynaptic neurotransmission, postsynaptic receptors, or both (DiAntonio et al., 1999). The signaling mechanisms underlying the interactions between the motor neuron and the muscle fiber in these homeostatic processes are complex and still not well understood (Ouanounou et al., 2016). It is known that the CAMKII and TOR pathways are necessary for neuromuscular homeostatic synaptic control (Haghighi et al., 2003; Penney et al., 2012), but many other potential signaling pathways remain understudied.

Early growth response-1 (Egr-1) is a transcription factor involved in a wide-variety of physiological and homeostatic processes. It mediates the direct regulation of many genes involved in homeostatic signaling and in the formation, maintenance and density of synapses (Gashler and Sukhatme, 1995; Li et al., 2005; Pagel and Deindl, 2011). Egr-1 is highly expressed in the brain where it regulates signaling pathways impacting learning, memory and synaptic plasticity (Jones et al., 2001; Penke et al., 2014). Indeed, Egr-1 regulates expression of the plasticity related activity-regulated cytoskeletal-related (Arc) gene (Li et al., 2005) while microarray studies have linked Egr-1 overexpression with changes in the expression of synaptic genes (James et al., 2005; Baumgärtel et al., 2009). In this particular study, a 65% reduction in *AGRN* gene expression was observed in rat PC12 neuroblastoma cells overexpressing Egr-1, and four putative Egr-1 binding sites were mapped to the *AGRN* gene promoter (James et al., 2005).

The *AGRN* gene encodes the heparan sulfate proteoglycan agrin, which mediates the formation, maintenance and stabilization of chemical synapses in both the brain and NMJ (McMahan, 1990; Gautam et al., 1996; Sanes and Lichtman, 1999). In the brain, agrin facilitates the establishment and functionality of excitatory synapses, is an important structural component of brain endothelial cell basement membranes forming the blood brain barrier, and may be important in development of neurological disease such as Alzheimer’s disease (Ferreira, 1999; Kröger and Schröder, 2002; Ksiazek et al., 2007; Rauch et al., 2011; Steiner et al., 2014). At the NMJ, agrin is critical for the organization, stabilization and maintenance of the postsynaptic apparatus. Homozygous agrin gene knockout is lethal due to a lack of functional NMJs (Gautam et al., 1996).

The agrin protein is cleaved at its proteolytic α- and β-sites by a serine protease known as neurotrypsin, releasing agrin cleavage fragments and rendering the agrin protein “inactive”. The agrin cleavage process influences agrin function in normal physiology as well as pathology, as NMJ maturation is dependent upon normal agrin cleavage and aberrant agrin cleavage is linked to neurological disease such as mental retardation and neuromuscular pathologies such as muscular dystrophy and sarcopenia (Bentzinger et al., 2005; Reif et al., 2007; Frischknecht et al., 2008; Bütikofer et al., 2011; Drey et al., 2013; Marzetti et al., 2014; Pribiag et al., 2014; Kalinkovich and Livshits, 2015; Landi et al., 2016). Importantly, the C-terminal 22 kDa agrin cleavage fragment (CAF) is increasingly being investigated as a biomarker of age-related sarcopenia (Feng and Ko, 2008; Lin et al., 2008a; Hettwer et al., 2013; Rudolf et al., 2014; Kalinkovich and Livshits, 2015; Landi et al., 2016). Thus, adequate and balanced expression and cleavage of agrin are important for proper synapse function, particular at the NMJ. Little is known, however, about regulation of *AGRN* gene expression.

As Egr-1 regulates synaptic plasticity in the brain and affects *AGRN* expression (James et al., 2005), we sought to investigate the relationship between Egr-1 and agrin and subsequent role in synaptic physiology. We hypothesized that Egr-1, through regulation of agrin expression, would mediate synaptic function at the NMJ. Importantly, Egr-1 is well known to be highly expressed in both vascular smooth and skeletal muscle (Pagel and Deindl, 2011). After establishing a direct relationship between Egr-1 and agrin expression in the brain, it was confirmed at the NMJ. Next, we investigated the functional involvement of the Egr-1-agrin pathway at the NMJ, where the role of agrin is crucial. The data from this study show that the Egr-1-agrin pathway influences morphological, electrophysiological and behavioral properties at the NMJ.

## Materials and Methods

### Animals

This study was carried out in accordance with the recommendations of the Canadian Council of Animal Care and the Animal Care Committee at the Lady Davis Institute. The protocol was approved by the Animal Care Committee at the Lady Davis Institute. Egr1−/− (knockout) C57 BL/6 mice (originally established by Dr Jeffrey Milbrandt) were bred and genotyped using standard methods (Lee et al., 1995). C57Bl/6J mice were used as controls (Stock No: 000664). All studies using dissected mouse brain, diaphragm and soleus muscle homogenates used for quantitative real time polymerase chain reaction (qPCR) and immunoblotting were performed on 3–5 month old wild type (WT) and Egr-1−/− mouse littermates. The *n* for each experiment is specified in “Results” Section.

### Cell Culture

BE (2)-M17 human neuroblastoma (M17) cells (Andres et al., 2013) and C2C12 mouse myoblasts were cultured as previously described (Yaffe and Saxel, 1977). Primary hippocampal neurons from post-natal day 0 (P0) Sprague–Dawley rat pups were isolated and cultured using standard methods including the addition of Ara-C (Jones et al., 2012). All cultures were maintained at 37°C in 5% CO_2_.

### Transfection

Human Egr-1 cDNA (OriGene) in a pCMV6-XL5 vector (Egr-1-pCMV6-XL5; Origene, Rockville, MD, USA) and control empty vector pCMV6 were used to manipulate Egr-1 overexpression. For transient transfections, cells at about 70%–80% confluency were transfected using Lipofectamine 2000 (Life Technologies, Burlington, ON, Canada) following the manufacturer’s protocol. Cells were harvested or fixed 24–48 h after transfection.

### Viral Transduction and Infection

Human myc-tagged Egr-1 cDNA (OriGene) was cloned into PLVX-IRES-ZsGreen1 (Clontech Laboratories, Inc., Mountain View, CA, USA), a bi-cistronic lentiviral vector with a CMV promoter to create PLVX-IRES-ZsGreen1-Egr-1. Empty PLVX-IRES-ZsGreen1 was used as vector control. Virus was produced using the Lenti-X™ HTX Packaging system (Clontech Laboratories Inc.). Viral titers ranged from 1 × 10^7^ to 1 × 10^8^ PFU/ml. To transduce neurons, virus was diluted in neuronal growth media, applied to cell monolayers for 6–24 h and then the medium was replaced. Primary neurons were infected at 10 days *in vitro* (DIV) or longer, with multiplicity of infection (MOI) ranging from 1 to 20.

### Immunoblotting

Cells were harvested in RIPA buffer (1 M Tris, pH 7.6, 5 M NaCl, 1% NP-40, 10% SDS, 0.5% w/v sodium deoxycholate, 0.5 M EDTA) containing a protease inhibitor cocktail (Roche, Laval, QC, Canada). Mouse tissue samples were collected and homogenized in Brain Homogenization Buffer (50 mM Tris, pH 7.2–7.4, 150 mM NaCl, 1% NP-40, 1 mM DTT, 5 mM EDTA) containing a phosphatase and protease inhibitor cocktail (Roche, Laval, QC, Canada). Quantification of protein used the Bio-Rad Protein Assay (Bio-Rad, Hercules, CA, USA). Immunoblotting was performed using standard methods (Mahmood and Yang, 2012). Proteins from SDS-PAGE were transferred to PVDF membrane (GE Healthcare, Mississauga, ON, Canada) and incubated with protein-specific antibodies overnight at 4°C: mouse monoclonal anti-β-actin (1:5000; Sigma, Oakville, ON, Canada), rabbit polyclonal anti-Egr-1 (1:1000; Santa Cruz Biotechnology, Dallas, TX, USA), mouse monoclonal anti-agrin (1:1000; Millipore Billerica, MA, USA), rabbit polyclonal anti-neurotrypsin (1:1000; Abcam, Toronto, ON, Canada). Blots were developed using Pierce ECL Plus Western Blotting Substrate (Thermo Scientific, Wilmington, DE, USA). For Western Blots using agrin antibody, PVDF membranes were stripped and reprobed, as agrin full length protein and cleavage fragments are spread between 220 kDa and 22 kDa. When applicable, stripping of the PVDF membrane for the purposes of re-probing was done using Restore Western Blot Stripping Buffer (Thermo Fisher Scientific, Cat. 21059) using the manufacturer’s protocol. Densitometry was quantified using ImageJ software (NIH, Bethesda, MD, USA), and values for each protein of interest were normalized to β-actin, GAPDH, or to the Coomassie stained membrane.

### Quantitative Real Time Polymerase Chain Reaction (qPCR)

Samples from transfected cells or harvested animal tissue were lysed in Qiazol (Qiagen, Toronto, ON, Canada). RNA was isolated using the RNeasy or RNeasy Lipid RNA extraction kit (Qiagen, Toronto, ON, Canada) following the instructions provided in the manufacturer’s protocol. One microgram of extracted RNA was reverse-transcribed using qScript™ cDNA SuperMix (Quanta Bioscience, Gaithersburg, MD, USA). mRNA expression was measured using a MicroAmp Fast Optical 96-well reaction plate (Life Technologies, Burlington, ON, Canada) and SYBRGreen (Qiagen, Toronto, ON, Canada) on an ABI 7500 Fast Real-Time PCR machine (Life Technologies, Burlington, ON, Canada). Agrin specific primers for qRT-PCR were purchased from (Qiagen, Toronto, ON, Canada). The following qPCR primer sequences were used for detection of human Egr-1 5′ ACCCCTCTGTCTTACTATTAAGGC and 5′ TGGGACTGGTAGCTGGTATTG; human GAPDH 5′ GTCTCCTCTGACTTCAACAGCG and 5′ ACCACCCTGTTGCTGTAGCCAA; and mouse β-actin 5′ AAGAGCTATGAGCTGCCTGA and 5′ TACGGATGTCAACGTCACAC. Fold changes were calculated using the ΔΔCT method with one of the aforementioned housekeeping genes as the endogenous control.

### Chromatin Immunoprecipitation (ChIP) Assay

M17 human neuroblastoma cells were transiently transfected with pcDNA3.1-Egr-1 plasmid for 48 h. chromatin immunoprecipitation (ChIP) was carried out using the EZ-Magna ChIP™ A-ChIP Kit (Millipore, Billerica, MA, USA) following the manufacturer’s protocol. Sonicated lysates were incubated at 4°C using rabbit polyclonal Egr-1 (Santa Cruz Biotechnology, Inc., Dallas, TX, USA) or rabbit IgG (Santa Cruz Biotechnology, Inc., Dallas, TX, USA) antibodies. The resulting purified DNA was amplified and quantified using real-time PCR. Agrin primer sets (P) and Negative Control (NC) primer sets were as follows: P1: Forward(AGCCAGGCCAAGCTGCAGAG), P1:Reverse(CCACGCCCCTTCCCAGCTC), P2: Forward(CTCGGCAGTCCCGTCACGTC), P2: Reverse(TGCACCCGAGTTAATGCCTAATCTGGA), P3: Forward(AGACAAGTGCACCTCGCCCA), P3: Revers(TTCTCGGCCGGTAGGGAGGG), P4: Forward(GGAAGGGGCTGCTTCTCCCG), P4: Reverse(GGACAGGGACCCACGCACTC), NC: Forward(CCCCAGGATGGGAGTGCCAA), NC: Reverse(GGCGGCCCTTGGTGAACTTG). Purified DNA from both Egr-1-DNA and IgG-DNA complexes were amplified using qPCR with the above listed primer sets. Data values from Egr-1-DNA complexes were divided by the values from IgG-DNA control complexes, and the highest value artificially set at 100. Statistical significance was calculated relative to the NC. All values were normalized to IgG.

### Diaphragm NMJ Analysis

Surgical laparotomy was performed on five WT and five Egr-1−/− mice at 4–6 months of age, and the thoracic cavity was removed (Martin et al., 2015). The diaphragm, still fully attached to the ribcage was placed in a 4% paraformaldehyde solution in PBS for 1 h, connective tissue removed, diaphragm muscles washed. Diaphragm tissue was incubated with tetramethylrhodamine α-bungarotoxin (Molecular Probes/Invitrogen) 25 μg/mL diluted 1:40 in PBS for 30 min, washed, treated with methanol and then whole mounted using glass coverslips and ClearVue™ Mountants (Thermoscientific).

### Analysis and Confocal Imaging

Real-time confocal microscopy analysis was used to image and analyze the entire diaphragm. Confocal images were obtained with a Leica DM LFSA microscope with an Ultraview confocal scanner (PerkinElmer). The images were captured and processed using Metamorph software (Universal Imaging). Starting from the beginning of one hemi diaphragm, consecutive images were taken without overlap to the end. Z-stack images with a depth encompassing the entire depth of the motor endplates were used. For each whole diaphragm, the sum of α-bungarotoxin (Btx) stained endplates was quantified using ImageJ. Also using ImageJ, the bandwidth of innervation was configured by measuring a series of straight lines drawn using the software from one side of the band to the other and averaged per genotype. The individual area was calculated by tracing the circumference of individual motor endplates. ImageJ quantification in pixels was converted to micrometer using standard conversion equations.

### Soleus NMJ Analysis

Soleus muscle fibers from five adult WT and five Egr-1−/− mice at 12–14 months of age were stained with Btx to view AchRs, and labeled with anti-myosin heavy chain (anti-MHC) antibodies to distinguish between muscle fiber types. NMJ immunohistochemical labeling was done as previously described (Arbour et al., 2015). Soleus muscles were first dissected in oxygenated Rees’ Ringer’s solution, secured in a Sylguard-coated 10 mm Petri dish, fixed for 10 min in 4% formaldehyde diluted in PBS buffer (in mm as follows: 137 NaCl, 2.7 KCl, 10 Na_2_HPO_4_, 2 KH_2_PO_4_) at room temperature, and permeabilized in 100% cold-methanol at −20°C for 6 min. Blocking was done by incubating muscles for 20 min at room temperature with 10% normal donkey serum in PBS containing 0.01% Triton X-100. To distinguish between type I and type II muscle fibers, muscles were incubated with mouse anti-Type I MHC IgGIIb (MHC-I; magenta) and anti-Type IIa MHC IgG1 (MHC-IIa; green; 1:100–200, SC-71-c and 1:75–100, BA-D5-c, Developmental Studies Hybridoma Bank, respectively) for 120 min at room temperature. After 3 × 5 min rinses with PBS, the muscle preparations were incubated with CY5 or Alexa-647 coupled anti-mouse IgGIIb (1:500) secondary antibodies for 60 min, and then incubated with Alexa-488 coupled anti-mouse IgG1 (1:1000) for 60 min at room temperature. Finally, to view AchRs, the muscles were incubated with αBtx (Alexa-594, 1.33–2.0 μg/ml) for 45 min, and the preparations were mounted in Prolong Gold anti-fade reagent. Confocal imaging was done using a Zeiss LSM 510 confocal microscope or the spectral detection feature of an Olympus FV1000. No manipulations of the images were performed after acquisition.

NMJ morphology (density of surface NMJs, percentage of smooth NMJs, percentage of faint endplates, number of fragments per endplate) were analyzed using criteria similar to those described previously (Wright et al., 2009; Samuel et al., 2012; Arbour et al., 2015).

### Muscle Electrophysiology

Electrophysiology experiments were carried out using soleus muscles isolated from WT and Egr-1−/− adult littermates between 12 months and 14 months of age, as previously described (Arbour et al., 2015). Twenty-one NMJs were recorded from five different WT mice, and 26 NMJs were recorded from five different Egr-1−/− mice. Stimulation of the tibial nerve was performed using a suction electrode filled with extracellular saline. Endplate potentials (EPPs) were recorded using glass microelectrodes (1.0 mm OD; WPI) pulled to 40–70 mΩ (filled with 3 mm KCl) with a Brown–Flaming micropipette puller (Sutter Instruments). Synaptic responses were amplified by an AM Systems 1600 amplifier and further amplified (100×) and filtered (2 kHz) by a Warner Instruments DC amplifier. The recordings were digitized using a national Instruments BNC 2110 board and subsequently acquired with WinWCP software (John Dempster, Strathclyde University, Strathclyde, UK).

Synaptic strength of recorded NMJs was determined by measuring the paired-pulse facilitation (PPF) and the quantal content (m). These were obtained using a low Ca^2+^ (1 mm) and high Mg^2+^ (6.4 mm) Ringer’s solution. PPF was obtained using two stimuli of 0.1 ms duration at 15 ms interval, elicited at 0.2 Hz. PPF was calculated as the mean amplitude of the second EPPs divided by the mean amplitude of the first EPPs, including failures. Quantal content (m) was determined using the amplitude of the miniature endplate potentials (MEPPs), as described previously (Del Castillo and Katz, 1954): m = (mean amplitude of EPPs/mean amplitude of the MEPPS). MEPP amplitude and frequency were determined using 5–10 min of recordings without motor nerve stimulation. A minimum of 100 MEPPs were used to calculate m for each NMJ. Recordings with an initial membrane potential depolarized ≥65 mV or with >5 mV variation from holding potential were not included for analysis. Muscle fibers were impaled thick sim 50–100 μm from the NMJ to be studied, avoiding mechanical distortion of the NMJ, yet providing a morphological landmark for finding the NMJ for *post hoc* morphological analysis.

### Behavior Testing

Behavior motor assays were carried out on four WT and four Egr-1−/− mice at 10–12 months of age at the Neurophenotyping Service Platform of the Douglas Mental Health University Institute, Montreal. Briefly, **Open field locomotor activity** was used to assess general locomotor activity. Animals were placed in a plexiglass box for habituation then locomotor activity was measured and recorded for 30 min by using the Versamax computer program, which permits automated recording of horizontal and vertical movement, as well as stereotypy. **Inverted grid** test was used to assess limb muscle strength and endurance and was carried out in accordance with established guidelines (Kraemer et al., 2010; Luong et al., 2011). All behavioral testing was performed within the same time window each day to control for natural circadian variation of physical activity patterns in the mice.

### Statistics

Means were calculated, and data are shown as the mean ± SEM. Statistical analysis consisted of Student’s *t* test (unpaired, 2-tailed) for comparison of two variables, or two-way analysis of variance (ANOVA) for comparisons of variation within and between two or more variables, and were performed using GraphPad Prism 6 Software. Significance was set at *p* < 0.05.

## Results

### Egr-1 Binds Directly to AGRN Gene Locus

**Figure 1:**
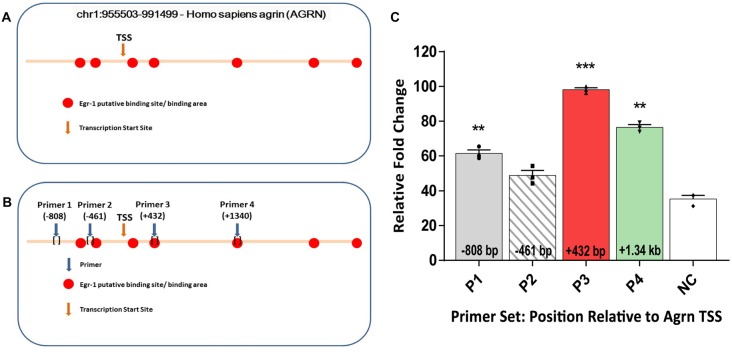
**(A)** *In-silico* analysis of human *AGRN* on chromosome 1 using the UCSC Genome Browser and Qiagen D-Code program identified several putative early growth response-1 (Egr-1) binding sites on the *AGRN* gene locus, both upstream and downstream of the TSS. **(B)** Primers were designed encompassing the putative binding sites, and four specific primer sets were used to amplify parts of the *AGRN* gene. **(C)** Chromatin immunoprecipitation (ChIP) assay was performed using these primer sets, and the Egr-1-*AGRN* interaction was determined using quantitative real time polymerase chain reaction (qPCR). Primers (P) 1, 3 and 4 showed binding activity, with the highest at region P3. Data was calculated as relative fold change, with the highest value set to 100. All results were normalized to internal levels of IgG and plotted relative to the binding activity of the negative control (NC) region from chromosome 1. *n* = 3. *p*-values were obtained using Student’s *t*-tests. *(*p* ≤ 0.05); **(*p* ≤ 0.01); ***(*p* ≤ 0.001); ****(*p* ≤ 0.0001).

To test the hypothesis that Egr-1 regulates *AGRN* gene expression in the brain, we performed *in-silico* analysis of the *AGRN* gene utilizing the Qiagen DECODE program and the USCS genome browser to search for Egr-1 putative binding sites. We identified seven putative Egr-1 binding sites on the *AGRN* gene locus (Figure 1A).

Based on the *in-silico* results, we tested whether Egr-1 bound directly to the human *AGRN* gene by performing ChIP using genomic Egr-1: DNA complexes extracted from human M17 neuroblastoma cells. Eleven sets of primers were designed to tile the Egr-1 putative sites, as well as two sets of primers several kilo-base pairs away served as NC. Regions P1 (*p* = 0.0082), P3 (*p* = 0.0004), and P4 (*p* = 0.0037, Figure 1B) showed significant amplification compared with the NC, with region P3 showing the highest amplification (Figure 1C). Thus, three of the primer sets demonstrated amplification that was above that of the NC, indicating that Egr-1 directly binds the *AGRN* gene locus at least at these three sites.

### Egr-1 Overexpression Decreases Agrin Protein Levels in Neuronal and Muscle Cell Lines

**Figure 2:**
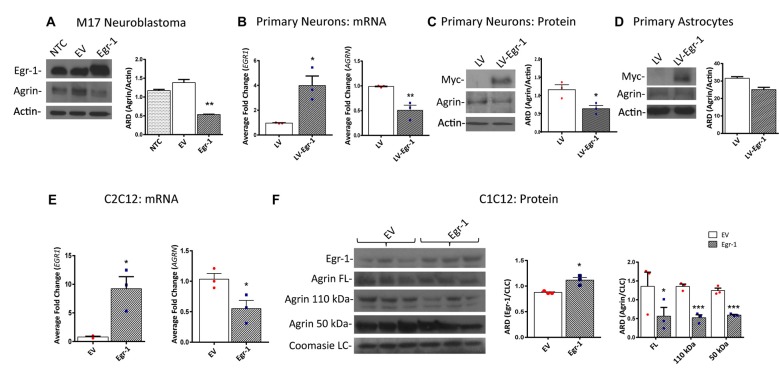
Human M17 neuroblastoma cells were transiently transfected with pcDNA3.1 and pcDNA3.1Egr-1. **(A)** Representative western blot from three independent experiments. Egr-1 overexpression led to a significant decrease in agrin protein expression with respect to controls (empty vector, EV). β-actin was used as a loading control. **(B)** Rat primary hippocampal neurons were infected at 11 days *in vitro* (11 DIV) with lentiviral control (LV) or lentivirus containing myc-tagged human Egr-1 (LV-Egr-1) at an multiplicity of infection (MOI) of 20. Overexpression of Egr-1 led to a significant increase in *EGR1* mRNA expression and decrease in *AGRN* mRNA expression with respect to LV controls. Values were normalized to GAPDH expression, and results were calculated using the ΔΔCT method and graphed as a fold-change of mRNA expression. *n* = 3. **(C)** Overexpression of myc-tagged LV-Egr-1 also led to a significant increase in myc and decrease in agrin protein expression. *n* = 3. **(D)** Next, rat primary hippocampal astrocytes were infected at 11 DIV with LV or LV-Egr-1 at an MOI of 20. Egr-1 overexpression did not significantly change agrin protein expression compared to LV controls in these cells. β-actin was used as a loading control. *n* = 3. **(E)** Mouse C2C12 myoblast cells were transiently transfected with pCMV6-XL5 (EV) and pCMV6-XL5-Egr-1(Egr-1). Overexpression of Egr-1 led to a significant increase in *EGR1* and decrease in *AGRN* mRNA levels with respect to EV controls. *n* = 3. **(F)** When Egr-1 was overexpressed in C2C12 cells, Egr-1 protein levels were increased, and full length, 110 kDa, and 50 kDa agrin protein expression were all significantly decreased compared to EV controls. Coomassie staining was used as a loading control. *n* = 4. *p*-values were obtained using Student’s *t*-tests or 2-way analysis of variance (ANOVA), respectively. *(*p* ≤ 0.05); **(*p* ≤ 0.01); ***(*p* ≤ 0.001); ****(*p* ≤ 0.0001).

Because ChIP analysis showed direct binding of Egr-1 to *AGRN*, we next tested the impact of Egr-1 overexpression on agrin protein expression *in vitro*. Agrin protein expression was compared using M17 human neuroblastoma cells transfected at ~70% confluency with empty vector or a plasmid expressing human Egr-1. Agrin protein expression was significantly reduced (*p* = 0.0094) in lysates prepared from Egr-1 overexpressing vs. empty vector transfected cells, suggesting that Egr-1 negatively regulates agrin expression (Figure 2A).

To establish this relationship in primary neuronal cultures, neurons were isolated from P0 rat hippocampi and infected with lentivirus overexpressing human Egr-1 or lentivector control (LV) at 11 DIV (MOI 20), and agrin expression was quantified. Egr-1 mRNA expression was significantly increased (*p* = 0.0164), and *AGRN* mRNA expression was significantly decreased (*p* = 0.0098) in neurons infected with LV-Egr-1 compared with LV (Figure 2B). To test this relationship at the protein level, Egr-1 and agrin protein levels were examined by immunoblotting. Here, when Egr-1 overexpression was detected by the presence of the myc-tag protein band, agrin protein expression was significantly decreased (*p* = 0.0281) in LV-Egr-1 compared with LV-infected cells (Figure 2C). Thus, Egr-1 overexpression in primary rat neurons reduced both agrin RNA and protein expression.

To investigate if the impact of Egr-1 on agrin expression was neuron specific, primary astrocytes at 11 DIV were infected with LV and LV-Egr-1 (MOI 20), and agrin protein expression measured. Agrin protein expression decreased, but did not reach significance (*p* = 0.0552) in LV-Egr-1 infected primary astrocytes compared with LV controls (Figure 2D), suggesting that increased Egr-1 expression does not robustly regulate agrin expression in astrocytes.

Since the role for agrin has been well characterized at the NMJ, the relationship between Egr-1 and agrin expression was tested in a muscle cell model. To test whether Egr-1 overexpression would reduce agrin expression in muscle cells, C2C12 myoblasts were transiently transfected with Egr-1-pCMV6-XL5 or empty vector (EV) at ~70% confluency, and then incubated in horse serum permitting myotube differentiation. *EGR1* mRNA was significantly increased (*p* = 0.0157) and *AGRN* mRNA was significantly reduced (*p* = 0.0422) in cells transfected with Egr-1-pCMV6-XL5 vs. EV (Figure 2E). Similarly, when Egr-1 protein levels were significantly increased (*p* = 0.0128) following transfection with Egr-1-pCMV6-XL5, agrin full length protein (*p* = 0.0498), as well as the 110 kDa (*p* = 0.001), and 50 kDa (*p* = 0.0007) agrin cleavage products were significantly decreased compared with EV transfected cells (Figure 2F).

Together, these data suggest that agrin protein expression is reduced as a result of increased Egr-1 expression, and that this effect results from regulation at the transcriptional level in human M17 neuroblastoma cells, rat primary neurons and mouse C2C12 myotubes.

### Agrin Expression Is Increased in Egr-1−/− Mouse Brain and Skeletal Muscle

**Figure 3:**
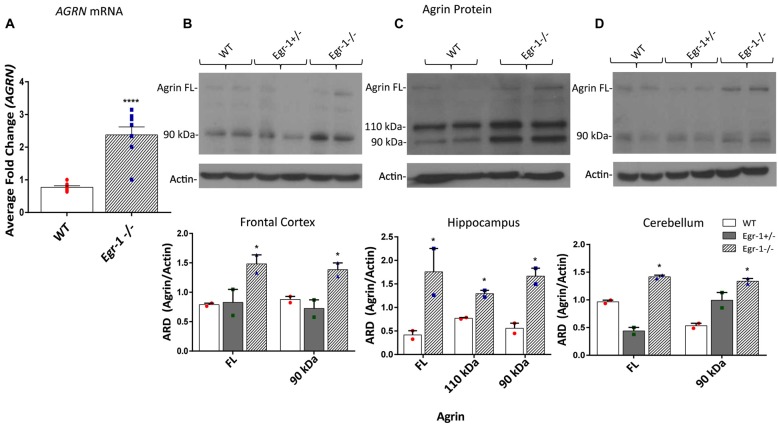
RNA was extracted from the brains of seven wild type (WT) and seven Egr-1−/− mice, and mRNA was isolated. **(A)** qPCR showed that Egr-1−/− mouse brain has significantly more *AGRN* mRNA expression compared with WT mice. Protein samples were prepared from the frontal cortex, hippocampus and cerebellum of six WT, six Egr-1+/− and six Egr-1−/− mouse brains. The western blots presented are representative blots from three independent experiments. **(B)** Expression of the full length and 90 kDa agrin were significantly increased in the Egr-1−/− frontal cortex compared with that of WT. **(C)** In the hippocampus, full length, 110 kDa and 90 kDa agrin expression were significantly increased in Egr-1−/− mouse brain compared with WT. **(D)** In the cerebellum, full length and 90 kDa agrin expression were significantly increased in Egr-1−/− mouse brain compared with WT. *p*-values were obtained using Student’s *t*-tests or 2-way ANOVA, respectively. *(*p* ≤ 0.05); **(*p* ≤ 0.01); ***(*p* ≤ 0.001); ****(*p* ≤ 0.0001).

Our *in vitro* data suggests that Egr-1 negatively regulates *AGRN* expression. To investigate whether this effect was found *in vivo*, RNA and protein samples were prepared from the brains of (WT, C57Bl/6J) and Egr-1−/− mice*. AGRN*-specific mRNA was significantly increased (*p* < 0.0001) in isolates prepared from whole brains of seven WT and seven Egr-1−/− mice (Figure 3A). To analyze agrin protein expression in specific brain areas, the hippocampus, frontal cortex, and cerebellum were dissected from the brains of six WT, six Egr-1+/−, and six Egr-1−/− mice and agrin protein expression was measured. The western blots presented in Figure 3 are representative blots from three independent experiments. Full length agrin protein (*p* = 0.0465) as well as the 90 kDa cleavage fragment (*p* = 0.0499) were significantly increased in homogenates from the frontal cortex (Figure 3B), hippocampus (FL *p* = 0.0423, 110 *p* = 0.019, 90 *p* = 0.0311, Figure 3C) and cerebellum (FL *p* = 0.0173, 90 *p* = 0.0469, Figure 3D) of Egr-1−/− compared with WT mice. Agrin expression was similar in Egr-1+/− and WT mice.

**Figure 4:**
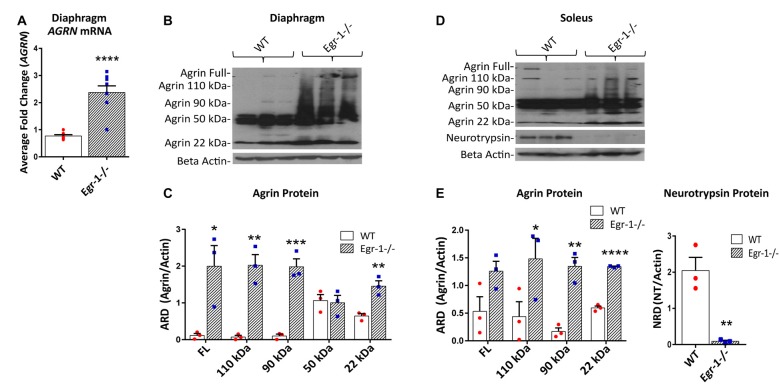
RNA and protein samples were prepared from nine WT and nine Egr-1−/− mouse diaphragm and soleus muscles. The western blots presented are representative blots from three independent experiments. **(A)** *AGRN* mRNA expression was significantly increased in Egr-1−/− mouse diaphragm when compared with WT. Similarly, in both diaphragm **(B,C)** and soleus muscles **(D,E)**, the expression of agrin was significantly increased in Egr-1−/− mice compared with WT, with the exception of full length agrin in the soleus, which did not reach significance. All agrin protein bands were normalized to β-actin. Protein expression of neurotrypsin in the soleus was significantly decreased in Egr-1−/− mice compared with WT. All neurotrypsin protein bands were normalized to β-actin. *p*-values were obtained using Student’s *t*-tests or 2-way ANOVA, respectively. *(*p* ≤ 0.05); **(*p* ≤ 0.01); ***(*p* ≤ 0.001); ****(*p* ≤ 0.0001).

To demonstrate an *in vivo* transcriptional relationship between Egr-1 and agrin expression in skeletal muscle, mRNA samples were prepared from the diaphragm and soleus of nine WT and nine Egr-1−/− mice. The western blots presented in Figure 4 are representative blots from three independent experiments. *AGRN* expression was significantly increased (*p* = *0* < 0.0001) in mRNA samples isolated from the diaphragm of Egr-1−/− vs. WT mice (Figure 4A). Similarly, agrin protein was significantly increased in both Egr-1−/− mouse diaphragm (full length, *p* = 0.029; 110 kDa, *p* = 0.0027; 90 kDa, *p* = 0.001; 22 kDa, *p* = 0.007; Figures 4B,C) and soleus muscles (110 kDa, *p* = 0.0464; 90 kDa, *p* = 0.0023; 22 kDa, *p* = 2.93 × 10^−5^; Figures 4D,E) compared to controls, with the exception of soleus full-length agrin which did not quite reach significance (full length, *p* = 0.051). Overall, these data suggest that an inverse relationship is present in multiple brain areas and in striated skeletal muscles *in vivo* such that agrin expression is increased when Egr-1 is absent.

Full length agrin cleavage by the enzyme neurotrypsin ultimately yields a 110, 90, and 20–22 kDa C-terminal agrin fragment (CAF). To determine whether the increase in CAF in the muscle in Egr-1−/− mice was due to an increase in the expression of neurotrypsin, protein levels of neurotrypsin were quantified in WT and Egr-1−/− mice soleus muscle tissue. Interestingly, neurotrypsin expression was significantly decreased (*p* = 0.006) in Egr-1−/− soleus muscle compared with WT (Figures 4D,E).

### NMJ Density, Endplate Area and Area of Innervation are Increased in Egr-1−/− Mice

**Figure 5:**
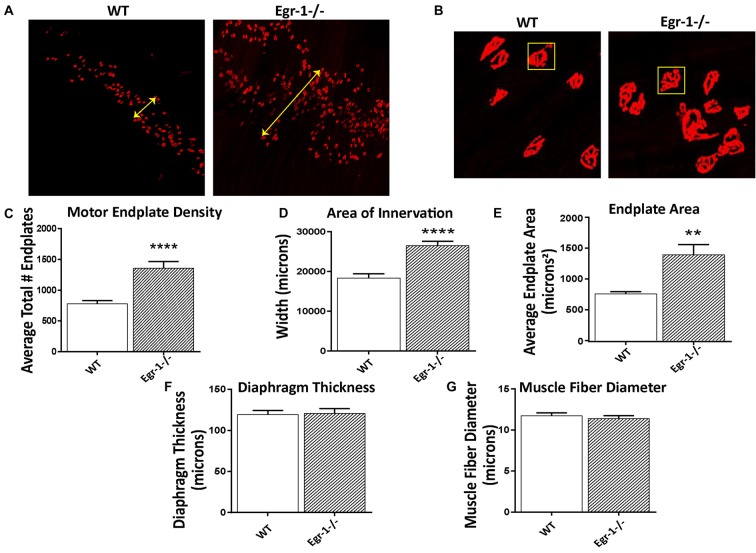
Diaphragms from five WT and five Egr-1−/− adult mice were whole mounted and stained with α-bungarotoxin to motor endplates made up of AchRs. **(A)** Representative 10× confocal images of WT and Egr-1−/− mouse diaphragm stained with α-bungarotoxin. **(B)** Representative 40× confocal images of WT and Egr-1−/− mouse diaphragm stained with α-bungarotoxin. The **(C)** density (total number) and **(D)** area of nerve innervation are significantly increased in Egr-1−/− compared with WT. The yellow arrows in the WT and Egr-1−/− panels highlight the width of the innervation band. **(E)** In Egr-1−/− mouse diaphragm, the area of individual endplates was significantly increased compared to WT. The size difference in the endplates is evident by comparing the identically-sized yellow boxes in the WT and Egr-1−/− panels. There was no significant difference in the thickness of the diaphragm, **(F)** or in the muscle fiber diameter, **(G)** in Egr-1−/− mouse diaphragm vs. WT. Z-stack images were quantified and analyzed to obtain results, and analysis was done using ImageJ software. *p*-values were obtained using Student’s *t*-tests. *(*p* ≤ 0.05); **(*p* ≤ 0.01); ***(*p* ≤ 0.001); ****(*p* ≤ 0.0001).

To investigate the impact of the inverse Egr-1-agrin relationship in synaptic function at the NMJ, the number and size of motor endplates in whole-mounted diaphragm muscle isolated from five WT and five Egr-1−/− were quantified. Diaphragm muscle tissues were stained with α-Btx to visualize AChRs. There was a significant increase (*p* < 0.0001) in the total number of α-Btx- labeled motor endplates in Egr-1−/− vs. WT mice (Figures 5A,C). Further, the area of innervation (*p* < 0.0001, Figures 5A,D) and the average endplate area (*p* = 0.0011, Figures 5B,E) were increased in Egr-1−/− compared with WT. To rule out that these increases were due to differences in the size of the diaphragm or muscle fibers, both parameters were quantified. No significant difference was found in the thickness of the diaphragm (*p* = 0.8752, Figure 5F) or in the muscle fiber diameter (*p* = 0.5091, Figure 5G), between WT and Egr-1−/− mice.

### Soleus NMJs of Egr-1−/− Mice Display Less Smooth, more Fragmented Endplate Structures

**Figure 6:**
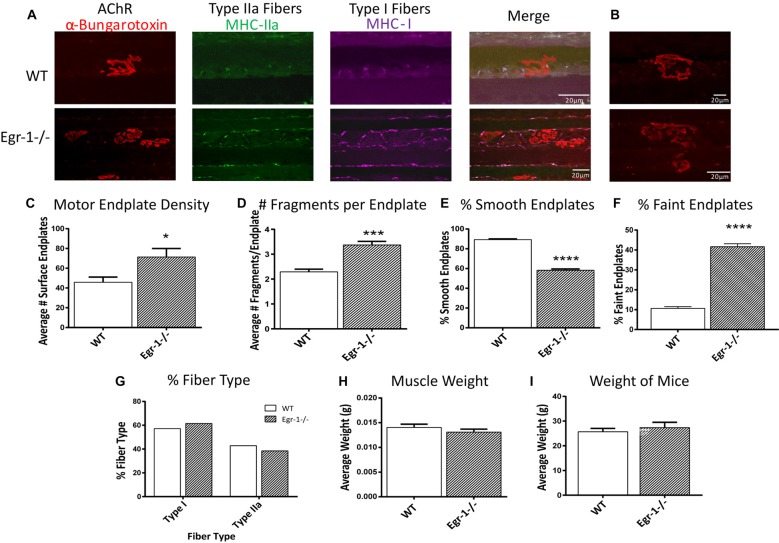
**(A,B)** Representative stacked confocal images of neuromuscular junctions (NMJs) from five WT and five Egr-1−/− mice soleus muscle NMJs. Soleus muscle preparations from WT and Egr-1−/− mice were labeled with anti-myosin heavy chain (anti-MHC) monoclonal antibodies to view Type I fibers (MHC-I; magenta), type IIa fibers (MHC-IIa; green), and then stained with α-bungarotoxin. *n* = 5. The mean number of surface endplates, **(C)** and NMJ fragments, **(D)** were increased in Egr-1−/− mice compared to WT. The % of smooth endplates **(E)** was decreased, and the % of faint NMJs was increased **(F)** in Egr-1−/− mouse soleus NMJs compared to WT. In WT vs. Egr-1−/− mice, there was no significant difference in the % of type I and type IIa soleus muscle fibers, **(G)** the weight of the soleus muscle, **(H)** or the overall bodyweight of the mice, **(I)** *p*-values were obtained using Student’s *t*-tests. *(*p* ≤ 0.05); **(*p* ≤ 0.01); ***(*p* ≤ 0.001); ****(*p* ≤ 0.0001).

Because of morphological differences in diaphragm NMJs in WT vs. Egr-1−/− mice (Figure 5), the NMJs of the soleus muscle were further analyzed and characterized (Wright et al., 2009; Samuel et al., 2012; Arbour et al., 2015). Soleus muscle preparations from five WT and five Egr-1−/− mice were labeled with subtype specific anti-MHC monoclonal antibodies to identify Type I fibers (MHC-I), and type IIa fibers (MHC-IIa), and with α-Btx to label motor endplates (Figures 6A,B). As was detected in the NMJs of the diaphragm, there was a significantly greater density of motor endplates (*p* = 0.015) in Egr-1−/− mouse soleus NMJs, with respect to WT (Figure 6C).

Soleus NMJs showed further evidence of disorganized endplate structure, known to be important for the NMJ function. First, quantification of the number of fragments per endplate revealed that soleus NMJs in Egr-1−/− mice were significantly more fragmented (*p* = 0.0003) than those of WT (Figures 6B,D). Next, the contour and edging of each endplate was qualified whereby endplates were designated as smooth when the edges were clear and distinct, or non-smooth when the edges were fuzzy or unclear. Egr-1−/− mice had a significantly lower percentage of smooth endplates (*p* < 0.0001) compared with WT mice (Figures 6B,E). Lastly, the postsynaptic sites of WT and Egr-1−/− mouse soleus NMJs that were noticeably faint and had a lack of organization, were characterized as “faint NMJs”, which are reminiscent of immature or disintegrating NMJs. Egr-1−/− mice had a significant increase (*p* = < 0.0001) in the percentage of faint NMJs when compared with WT (Figures 6B,F). The differences between WT and Egr-1−/− mouse NMJs were not due to a difference in the percentage of slow- or fast-twitch muscle fibers, the weight of the soleus muscle (*p* = 0.3072), or the overall body weight (*p* = 0.5522) of the mice (Figures 6G–I).

### Soleus NMJs of Egr-1−/− Mice Display Increased Quantal Content

We next tested whether functional synaptic alterations were observed in addition to the morphological alterations described above. Diaphragm and soleus muscles are composed primarily of slow twitch (Type I) muscle fibers (Polla et al., 2004). Hence, the soleus was chosen to represent both muscle types. Twenty-one and 26 NMJs were analyzed from five WT mice and five Egr-1−/− mice, respectively.

**Figure 7:**
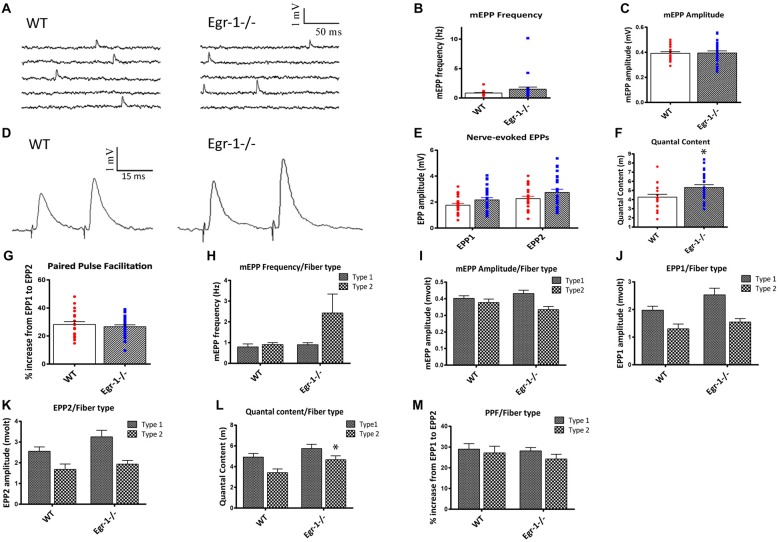
Soleus muscle preparations from WT and Egr-1−/− mice were subjected to electrophysiological recordings. Twenty-one NMJs were recorded from five different WT mice, and 26 NMJs were recorded from five different Egr-1−/− mice. **(A)** Representation of spontaneous miniature endplate potential (MEPP) recordings from WT and Egr−/− mouse soleus NMJS. There was no significant difference in the mEPP frequency, **(B)** or amplitude, **(C)** in WT vs. Egr-1−/− mouse soleus NMJs. **(D)** Representation of Endplate potentials (EPPs) evoked by paired-pulse stimulation of the motor nerve (15 ms interval) from WT and Egr-1−/− mouse soleus NMJs. The EPP amplitude was similar in Egr-1−/− and WT mice, **(E)** but Egr-1−/− mice displayed an increase in quantal content **(F,G)**. There was no significant difference in the paired-pulse facilitation (PPF) between genotypes. To analyze the electrophysiology results according to fiber type, each NMJ recording was sorted according to fiber type and analyzed. There were no significant differences in the mEPP frequency, **(H)** or amplitude, **(I)** between genotypes in type I or type IIa fibers. There was no significance difference in EPP amplitude in either fiber type **(J–L)**, although the quantal content was increased in type IIa fibers **(L,M)**. There was no significant difference in PPF between genotypes when sorted by fiber type. *p*-values were obtained using Student’s *t*-tests or 2-way ANOVA, respectively. *(*p* ≤ 0.05); **(*p* ≤ 0.01); ***(*p* ≤ 0.001); ****(*p* ≤ 0.0001).

First, spontaneous events were recorded, as shown in Figure 7A. There were no significant differences in mEPP frequency (*p* = 0.1454) or amplitude (*p* = 0.9040 (Figures 7A–C). This suggests that presynaptic events underlying spontaneous activity occur normally and that the postsynaptic receptors are not altered in the Egr-1−/− mice. Next, the motor nerve was stimulated via paired-pulse stimulation with a 15 ms interval, and EPP amplitudes were measured (Figure 7D). The EPP amplitude was similar (*p* = 0.054) in Egr-1−/− mice when compared with WT (Figure 7E). However, we observed a larger quantal content when compared with WT mice (*p* = 0.019, Figure 7F). There was no significant difference in the PPF (*p* = 0.51) between genotypes (Figure 7G). Thus, the increase in quantal content suggests that Egr-1−/− mouse NMJs exhibit enhanced synaptic transmission in comparison with WT.

As expected of a slow twitch muscle, there was no significant difference in muscle fiber type, as soleus NMJs from both WT and Egr-1−/− had more type I than type IIa fibers, Figure 6G. To analyze the electrophysiology results according to fiber type, each NMJ recording was sorted according to their fiber typing (type I vs. type IIa). There was no significant difference in the mEPP frequency (type I *p* = 0.535; type IIa *p* = 0.16) or amplitude (type I *p* = 0.295; type IIa *p* = 0.154) between genotypes in type I or type IIa fibers (Figures 7H,I). There was no significant difference in EPP amplitude in each fiber type (type I *p* = 0.084; type IIa *p* = 0.265 Figures 7J,K), although the quantal content was increased in type IIa fibers (type I *p* = 0.161; type IIa *p* = 0.027, Figure 7L). Similar to Figure 7F, there was no significant difference in PPF between genotypes when sorted by fiber type (type I *p* = 0.784; type IIa *p* = 0.455, Figure 7M). Together, these data indicate that Egr-1−/− mice have enhanced NMJ synaptic transmission which appears to be mainly presynaptic in origin, particularly targeting type IIa fibers.

### Do Egr-1−/− Mice Display Any Motor Defects?

**Figure 8:**
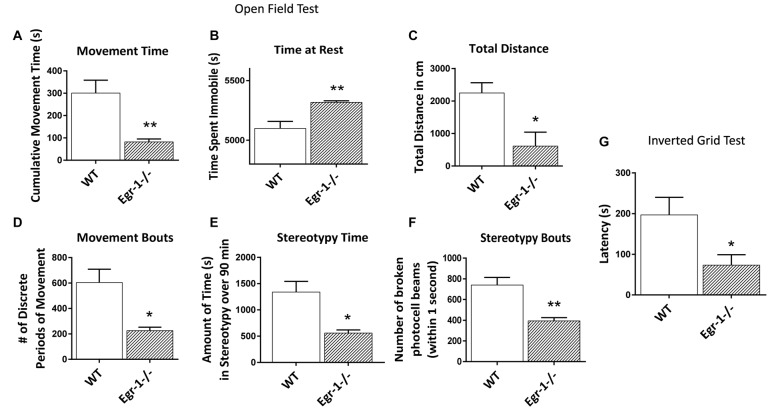
Four WT and four Egr-1−/− littermate adult mice were subjected to the open field test to measure general locomotion. The time spent moving, **(A)** was decreased in Egr-1−/− compared with WT mice and time spent at rest, **(B)** was increased. The Egr-1−/− moved a total distance that was less than WT mice, **(C)** and showed a decrease in the number discrete movement bouts, **(D)** the time spent in stereotypy movement, **(E)** and in discrete bouts of stereotypy, **(F)** when compared with WT mice. The same mice were subjected to the inverted grid test to measure strength and endurance in limb muscle. **(G)** Egr-1−/− 7458 show a significant decrease in limb strength and endurance compared to WT mice, as shown by the average latency in seconds that it took for the mice to fall from the inverted grid. *p*-values were obtained using Student’s *t*-tests. *(*p* ≤ 0.05); **(*p* ≤ 0.01); ***(*p* ≤ 0.001); ****(*p* ≤ 0.0001).

Due to the differences seen in NMJ morphology and in muscle electrophysiology in Egr-1−/− compared with WT, we tested whether Egr-1−/− mice would have any functional motor defects. Motor tests were done in four WT and four Egr-1−/− mice. In the open field test used to measure general locomotor activity, Egr-1−/− mice spent significantly less time moving (*p* = 0.01), and significantly more time immobile (*p* = 0.01) compared with WT mice (Figures 8A,B). Furthermore, Egr-1−/− mice covered shorter distances (*p* = 0.02; Figure 8C) and had fewer movement bouts (*p* = 0.013, Figure 8D) than WT mice. Stereotypy is defined as persistent, repetitive bouts of movement. Egr-1−/− mice spent significantly less time in stereotypy (*p* = 0.011), with fewer stereotypy bouts (*p* = 0.005) in comparison with WT mice (Figures 8E,F). In the inverted grid test, Egr-1−/− mice were unable to maintain their grip on the inverted grid as long as WT mice, and displayed a significant decrease (*p* = 0.04) in the latency to fall from the grid (Figure 8G). Interestingly, one of the four Egr-1−/− mice fell immediately in each of the trials. These data indicate that Egr-1−/− mice move less and have decreased muscle weakness and strength compared with WT mice.

## Discussion

In this study, we provide evidence that Egr-1 directly binds to and suppresses *AGRN* gene expression in cell lines derived from human, rat, and mouse, indicating significant conservation in mammals and species independency. Furthermore, this observation was confirmed in the brain and in at least two striated muscles *in vivo*. In addition to influencing agrin expression, this study also establishes the involvement of Egr-1, either directly or indirectly, in the process of agrin cleavage. Finally, we investigated the potential functional involvement of the Egr-1-agrin pathway at the NMJ, where the role of agrin is critical and well understood.

Results from this study show that changes in the expression of Egr-1 influence morphological, electrophysiological and behavioral properties of the skeletal NMJ, possibly through the Egr-1-agrin pathway. Mice deficient in Egr-1 show an increase in endplate density, size, innervation area, and fragmentation, as well as an increase in acetylcholine release at the NMJ. Furthermore, a decrease in skeletal muscle limb strength and endurance was observed in correlation with the Egr-1-mediated NMJ defects. The data from this study unravel a novel pathway between Egr-1 and agrin, providing insight on regulation of *AGRN* expression and potential homeostatic mechanisms at the NMJ.

### Agrin and NMJ Integrity

The presence of fully functional agrin is required for integrity and efficacy of NMJ function. Previous studies show that treatment with agrin increases the number of postsynaptic specializations *in vitro*, and is sufficient to activate postsynaptic apparatus formation in denervated muscles (Cohen et al., 1997; Bezakova and Lømo, 2001; Bezakova et al., 2001). Many studies have also shown the importance of functional agrin *in vivo*, in the facilitation of synaptic signaling and stabilization (Gautam et al., 1996; Lin et al., 2008b; Wu et al., 2010; Bogdanik and Burgess, 2011; Barik et al., 2014). Complete deficiency of agrin is lethal in mice, as they die at birth due to embryonic failure of NMJ formation (Gautam et al., 1996; Bogdanik and Burgess, 2011).

Changes in the expression of particular agrin isoforms greatly affect the integrity and organization of the NMJ (Burgess et al., 2000). A small neuronal agrin peptide insert at the C-terminal “z” site (z+ neuronal agrin) is necessary for its synaptogenic function in the CNS and at the NMJ, as agrin isoforms without this insert have little or no synaptogenic ability (Gesemann et al., 1996; Burgess et al., 2000). Importantly, mutant mice lacking z+ neuronal agrin have decreased motor endplate density as well as abnormal intramuscular nerve branching and presynaptic differentiation (Gautam et al., 1996; Burgess et al., 2000; Samuel et al., 2012). When agrin is cleaved by neurotrypsin, the c-terminal fragment containing this insert is released, and synaptogenic function is lost. Mice that overexpress the agrin-cleaving enzyme neurotrypsin in motor neurons (SARCO mice) display increased rates of agrin cleavage and evident NMJ deterioration (Bolliger et al., 2010; Bütikofer et al., 2011).

Agrin expression is known to be increased via contact-mediated processes, action potential-dependent neurotransmission and neural activity (O’Connor et al., 1995; Lesuisse et al., 2000). Although, Egr-1 overexpression was correlated with decreased *AGRN* expression, among several other synaptic proteins, evidence for direct regulation was lacking (James et al., 2005). Data from this study showing an Egr-1-agrin interrelationship, together with the results of other studies, suggest that agrin expression and cleavage must be tightly controlled for stable and functional NMJs.

Egr-1−/− mice display both an increase in agrin expression and cleavage in addition to NMJ abnormalities including increased density and area of motor endplates and increased endplate fragmentation. Since motor endplate density and maturity are influenced by agrin expression and cleavage, the increases of either agrin expression or cleavage, or both combined, in the Egr-1−/− mice compared with WT may contribute to the endplate morphology differences. It is not known how much of the agrin that is increased in the muscle contains the z-insert, giving agrin the synaptogenic function. Greater synaptogenic agrin may promote NMJ stabilization, but greater cleavage may promote deterioration. Future studies to explore and investigate agrin protein expression and cleavage in detail are needed.

Changes in agrin expression and/or function ultimately affect subsequent agrin signaling. At the NMJ, agrin mediates a well-established pathway consisting of low-density lipoporotein receptor 4 (Lrp4) and muscle-specific kinase (MuSK), which overall governs the assembly and stabilization of the postsynaptic apparatus (Gesemann et al., 1996; Gillespie et al., 1996; Apel et al., 1997; Glass and Yancopoulos, 1997; Hoch, 1999; Lin et al., 2001; Wang et al., 2006). Interestingly, the patterning of motor endplates seen in the Egr-1−/− mice is similar to that found in mice with a high amount of active MuSK, both of which are seemingly reflective of increased agrin expression or signaling (Kim and Burden, 2008).

### Agrin Cleavage, NMJ Deterioration and Muscle Weakness

Muscle weakness can occur from physical muscle damage (Roubenoff and Hughes, 2000; Deschenes, 2004; Kalinkovich and Livshits, 2015), abnormal firing of NT from the motor neuron, or normal firing with aberrant neuromuscular connection (Deschenes et al., 2010; Rudolf et al., 2014). Importantly, a subset of sarcopenic patients have increased circulating CAF, a degradation product of agrin proteolysis, which suggests that increased agrin cleavage is a clinical feature of age-related sarcopenia (Landi et al., 2016). Egr-1−/− display a significant increase in all agrin cleavage fragments, and most relevantly the 22 kDa fragment was increased in the soleus muscle, compared to WT.

Currently, the serine protease neurotrypsin is the only characterized protease known to cleave agrin. Interestingly, the expression of neurotrypsin in Egr-1−/− mice was decreased in soleus muscle, but not in the brain, which may suggest that another protease is responsible for agrin cleavage in skeletal muscle. This corroborates previous findings that agrin cleavage in muscle and NMJ maturation were normal in neurotrypsin-deficient mice, suggesting that agrin cleavage is mediated by another protease in those mice (Bütikofer et al., 2011). Therefore, it is possible that agrin is cleaved by one or more proteases in skeletal muscle, or that a particular protease is increased to compensate for increased expression of agrin, leading to these effects. Thus, identification and investigation of potential agrin-cleaving proteases other than neurotrypsin may be important for the understanding of the data from this study, how agrin cleavage may be increased in the absence of neurotrypsin, and how agrin cleavage may be increased in associated skeletal muscle conditions such as sarcopenia (Reif et al., 2007; Stephan et al., 2008; Matsumoto-Miyai et al., 2009). Regardless of how agrin is cleaved in Egr-1−/− mice, the increased amount of cleavage fragments in both brain and muscle tissue suggest that cleavage is greatly increased with respect to WT mice.

One mouse model of sarcopenia, known as SARCO mice, overexpress neurotrypsin in motor neurons and therefore also have significantly increased agrin cleavage. These mice show increased agrin fragments including the 22 kDa CAF, as well as accelerated NMJ maturation and disintegration, muscle degeneration, and weakness (Bütikofer et al., 2011). In addition to increased agrin 22 kDa CAF levels in the soleus, Egr-1−/− mice also display signs of NMJ disintegration like the SARCO mice. Endplates in Egr-1−/− mouse soleus NMJs were significantly more faint and fragmented with respect to WT mice. It is well understood that NMJs gradually change form with age, and become increasingly fragmented due to the presence of many isolated regions of synaptic differentiation (Willadt et al., 2016). The fact that these observations were specific to soleus and did not occur in the diaphragm also corroborates findings from previous research that soleus NMJs are affected before those of diaphragm in the aging process (Willadt et al., 2016). On the other hand, the soleus muscle was analyzed at a later age than the diaphragm muscle, which may be a simple explanation for the reason the diaphragm did not show visual fragmentation like the soleus.

It has been suggested that fragmentation and endplate reorganization is associated with impairment of neuromuscular transmission, contributing to age-related muscle weakness (Banker et al., 1983; Bromberg and Scott, 1994; Trontelj and Stålberg, 1995). A mouse model associated with muscular dystrophy known as *mdx* mice display endplate reorganization similar to that of Egr-1−/− mice, with an increase endplate number, area, and area of the nerve (Pratt et al., 2015). Both the Egr-1−/− mice used in this study and the SARCO mice show significant limb muscle weakness, which is an early sign of sarcopenia and one of the main causes of increased disability and declined mobility in the elderly (Bolliger et al., 2010; Bütikofer et al., 2011).

Egr-1−/− mice showed a significant decrease in mobility in the open field test. Previous studies with Egr-1−/− mice reported normal (Jones et al., 2001), or only slight immobility in the open field test, which was attributed to increased anxiety levels (Ko et al., 2005). Anxiety levels, however, were not addressed in our study. Our data show that Egr-1−/− mice also had decreased limb muscle strength and endurance in the inverted grid test. The reduced mobility in conjunction with decreased limb muscle strength suggest a possibility of abnormal neurotransmission or aberrant neuromuscular connection.

### Egr-1 Regulates Mechanisms of Synaptic Function and Compensation at the NMJ

The process of aging is characterized by homeostatic synaptic alteration and compensation in both interneuron and neuromuscular synapses (Takamori, 2012, 2017). It is known that quantal content and endplate size increase proportionally with age (Banker et al., 1983; Everett and Ernst, 2004; Pousinha et al., 2012; Rocha et al., 2013). Furthermore, it is known that increased quantal content and larger NMJs are compensatory mechanisms and signs of premature aging (Deschenes, 2011; Rudolf et al., 2014). Electrophysiological recordings of Egr-1−/− mouse soleus NMJs showed increased neurotransmission in Egr-1−/− mouse NMJs compared with WT, as suggested by increased quantal content not quantal size.

Quantal content is a presynaptic marker of synaptic function and neurotransmitter release mechanisms, while quantal size is a reporter of postsynaptic receptor changes in muscle fibers in response to the neurotransmitter. The lack of changes in mEPP amplitude in Egr-1−/− mice suggests that the quantal size (i.e., vesicular ACh concentration) is unaffected. This suggests that there are more ACh vesicles released from each junction, but that there is no change in vesicle size or content. Interestingly, quantal content was increased primarily in fast-twitch, type IIa fibers, which is the fiber type known to be preferentially affected in comparison with type I fibers in aging and in disease (e.g., ALS), and changes in these fibers occur at earlier stages (Deschenes, 2004; Rowley et al., 2007; Wilson et al., 2012).

Egr-1−/− mice show an increased endplate density, endplate area and area of innervation at the NMJ. Endplate size can increase as a result of increased muscle fiber size, number, or response. Egr-1−/− mice, however, show no difference in muscle weight or makeup of muscle fiber type, suggesting that the larger endplate size may be related to a stronger muscle fiber response. It is possible that endplate size in Egr-1−/− mice is increased to compensate for increased quantal release or for downstream events. Alternatively, quantal content may be increased as a compensatory response to the larger endplate size. It is also possible that there is no significant increase in EPPs in the Egr-1−/− in response to the increased quantal content because the overall function of the endplates has begun to decrease, and the increased quantal content is compensating for an endplate with decreased efficiency. This may be a homeostatic effect, just as increased motor endplate size is a homeostatic effect of aging. Further studies to investigate this area and test homeostatic and compensatory synaptic mechanisms in Egr-1−/− compared with WT are needed.

Increased number, area and area of innervation seen in the NMJs of Egr-1−/− mice may occur for a number of reasons. First, these increases may occur as a direct result of the mice having greater expression of agrin. Alternatively, it has been demonstrated that axonal/nerve sprouting occurs with age (Rosenheimer, 1990; Santos and Caroni, 2003), leading to increased endplate density as well as fiber denervation and re-innervation (Banker et al., 1983; Fahim, 1997), which also occurs with aging (Thomson, 1957; Ribchester et al., 2004).

Thus, Egr-1 deficient mice display several characteristics that are suggestive of aging and reflective of homeostatic mechanisms associated with aging. However, there are still several unknowns. It is unknown if the NMJ releases more acetylcholine to compensate for degenerating muscle fibers. It is also unknown if the increase in agrin cleavage is due to the increase in agrin expression, and if the increase in endplate density is due to the increase in agrin expression or another factor such as increased synaptic branching. It is unknown if the area of nerve innervation is greater simply because there are more endplates, or because there is more innervation as a response to decreased function of existing endplates. While many of these questions remain unanswered, it is clear that Egr-1 signaling is involved and that changes in Egr-1 function affect agrin expression and cleavage. It does not, however, rule out the involvement of other Egr-1 targets and pathways mediating these same effects, including regulation of agrin expression.

### Transcription Factor Egr-1: Expression and Target Genes

Egr-1 is a transcription factor expressed highly in both neurological and neuromuscular systems (Beckmann and Wilce, 1997; Fromm and Rhode, 2004). Importantly, levels of Egr-1 have been shown to decrease with normal age, and to both increase and decrease under different pathological conditions, making the Egr-1−/− mice a potentially interesting model to study the effects of aging following this study, in particular to help understand the mechanisms of synaptic compensation and the physiology that are evident (Desjardins et al., 1997; Gómez Ravetti et al., 2010; Bartolotti et al., 2016).

While it is clear that Egr-1, likely through regulation of agrin expression, has a significant effect on NMJ function, it cannot be concluded that all of the observations noted throughout this study are influenced by the Egr-1-agrin pathway. Egr-1 is activated rapidly in response to many different factors, including neural activity and neurotransmission, growth factors, cytokines, pharmacological agents, stimulation, stress, and DNA damage, among others, indicating that Egr-1 is involved in a multitude of physiological processes and regulates many different synapse-associated genes (Gashler and Sukhatme, 1995; Beckmann and Wilce, 1997; O’Donovan et al., 1999; Clayton, 2000; James et al., 2005; Pagel and Deindl, 2011). In addition to agrin regulation as described in this study, Egr-1 also influences the expression and regulation of the plasticity related Arc gene, as well as SNARE, synapsin II, serpine I, galectin-3 binding protein, integrin associated protein (IAP), cystatin c, PSD95, Shank3, neuronal specific septin 3, s100B, phospholipase C, protein kinase C, BACE1 and drebrin among several others (James et al., 2005; Li et al., 2005; Baumgärtel et al., 2009; Qin et al., 2016; Cho et al., 2017). Thus, Egr-1 may have direct targets in addition to, or other than, agrin, which may contribute to or cause the noted changes of Egr-1−/− mice compared with WT, as seen in this study.

## Conclusion

**Figure 9:**
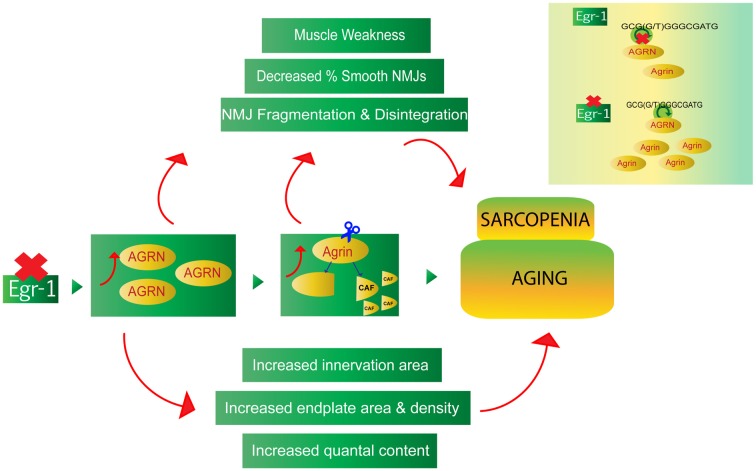
The Egr-1-agrin pathway may aid in the understanding of synaptic homeostatic mechanisms at the NMJ, which occur during the process of normal aging, as well as in the age-related condition known as sarcopenia. Reduction of Egr-1 expression leads to increased expression and cleavage of agrin in both brain and muscle as well as NMJ abnormalities, many of which are consistent with and reflective of aging and sarcopenia. Thus, dysregulation of the Egr-1-agrin pathway may provide a novel model system to study synaptic homeostasis at the NMJ, and Egr-1 deficient mice may serve as a suitable model to elucidate mechanisms associated with the underlying physiology of aging.

This study provides evidence of a novel Egr-1- agrin pathway with potential implications in homeostatic synaptic mechanisms at the NMJ. The data suggest that reduction of Egr-1 function leads to increased expression and cleavage of agrin in both brain and muscle, as well as NMJ abnormalities that are consistent with and reflective of age-related homeostatic and compensatory mechanisms (Figure 9). While future *in vivo* studies are needed to confirm direct binding of Egr-1 to *AGRN in vivo* and to thoroughly investigate the physiological implications of the Egr-1-agrin pathway and homeostatic mechanisms, Egr-1 deficient mice may serve as a novel model system to study synaptic physiology at the NMJ. As direct binding of Egr-1 to agrin was first established in a cell line derived from the CNS, and Egr-1 and agrin are both highly expressed and have been established as important synaptic mediators in the brain, future investigation of the Egr-1-agrin relationship in the central nervous system may be similarly interesting. Because Egr-1 governs many different pathways and sole manipulation of Egr-1 would be difficult, the Egr-1 deficient mice used in this study serve as a good model to investigate downstream targets of Egr-1, such as agrin, and the resulting signaling pathways affected that govern the physiological mechanisms of the synapse.

## Author Contributions

All listed authors have provided substantial contributions to the conception and design of this work, have participated in acquisition (RM, SB-A, AM, HP, JS), analysis (RM, SB-A, CC, SC, RR, HP, LEC), or interpretation of data (RM, SB-A, CC, SC, RR, HP, LEC). All listed authors have contributed to the drafting or critical revision of this work, have approved of this version of the article to be published, and have agreed to be accountable for all aspects.

## Conflict of Interest Statement

The authors declare that the research was conducted in the absence of any commercial or financial relationships that could be construed as a potential conflict of interest.
